# Association between dextromethorphan/bupropion with diabetes mellitus and hyperglycemia: reports to the Food and Drug Administration Adverse Event Reporting System (FAERS)

**DOI:** 10.1017/S1092852926101163

**Published:** 2026-08-07

**Authors:** Yang Jing Zheng, Sabrina Wong, Gia Han Le, Angela T. H. Kwan, Christine E. Dri, Roger Ho, Taeho Greg Rhee, Hernan F. Guillen-Burgos, Roger S. McIntyre

**Affiliations:** 1 Brain and Cognition Discovery Foundation, Canada; 2 University of Toronto Institute of Medical Science, Canada; 3 University Health Network, Canada; 4 University of Toronto Department of Pharmacology & Toxicology, Canada; 5 University of Ottawa Faculty of Medicine, Canada; 6 National University of Singapore Yong Loo Lin School of Medicine, Singapore; 7 National University of Singapore Institute for Health Innovation & Technology, Singapore; 8 The Hong Kong University of Science and Technology School of Science Division of, Hong Kong; 9 Psychiatry, Yale University School of Medicine, USA; 10 University of Connecticut School of Medicine, USA; 11 Department of Psychiatry and Mental Health, Pontificia Universidad Javeriana, Colombia; 12 Center for Clinical and Translational Research, Universidad Simon Bolivar, Venezuela; 13 Department of Psychiatry, University of Toronto, Canada

**Keywords:** Dextromethorphan/bupropion, auvelity, diabetes mellitus, hyperglycemia, metabolic condition, antidepressants

## Abstract

Dextromethorphan/bupropion (DXM/BUP) is the first orally administered antidepressant combining dextromethorphan, a non-competitive N-methyl-D-aspartate (NMDA) receptor antagonist and sigma-1 receptor agonist, and bupropion, a CYP2D6 inhibitor and antidepressant. DXM/BUP was approved by the United States Food and Drug Administration for Major Depressive Disorder (MDD) in adults in August 2022. Given that select psychotropic agents prescribed for MDD may alter glucose and insulin homeostasis, we investigated whether DXM/BUP was associated with disproportionate reporting of diabetes mellitus or hyperglycemia. Disproportionality analyses were conducted using reporting odds ratios (RORs), with DXM/BUP and comparator agents evaluated relative to a reference agent (acetaminophen). Data were obtained from the Food and Drug Administration Adverse Event Reporting System (FAERS) from the year of approval to April 26, 2026. A sensitivity analysis was performed on a restricted dataset from January 1, 2022, to April 30, 2026. Statistical significance was defined as a 95% confidence interval (CI) excluding 1.0. DXM/BUP was not associated with disproportionate reporting of diabetes mellitus (ROR = 0.33, 95% CI [0.07, 1.65], *p* = 0.18) or hyperglycemia (ROR = 0.10, 95% CI [0.01, 1.67], *p* = 0.11) relative to acetaminophen. Findings from the restricted 2022–2026 analysis were directionally consistent with the primary analysis. Given the sparse number of reports and wide CIs, these findings should be interpreted as preliminary and underpowered, rather than evidence of metabolic neutrality. Continued pharmacovigilance and prospective investigation are needed to further characterize the metabolic profile of DXM/BUP.

## Introduction

Dextromethorphan/bupropion (DXM/BUP) is the first oral antidepressant that combines (i) a non-competitive N-methyl-D-aspartate (NMDA) receptor antagonist and sigma-1 (σ-1) receptor agonist (dextromethorphan) with (ii) a CYP2D6 inhibitor and antidepressant (bupropion).^1^ Mechanistically, bupropion increases the bioavailability and half-life of dextromethorphan by inhibiting its metabolism, thereby maintaining therapeutic plasma concentrations.^2^ DXM/BUP was approved by the United States Food and Drug Administration (U.S. FDA) on August 18th, 2022, for the treatment of adults with Major Depressive Disorder (MDD).^1^ ^,^^3^ ^,^^4^ The emergence of DXM/BUP as a novel therapeutic for persons with MDD provides opportunities to assess tolerability and safety with pharmacovigilance reporting, particularly regarding metabolic concerns.

Given this context, it is pertinent to recognize that individuals living with MDD and bipolar disorder (BP) exhibit heightened vulnerability to metabolic dysregulation, including diabetes mellitus and hyperglycemia.^5^ ^,^^6^ Furthermore, several antidepressants and adjunctive therapies (eg, olanzapine, fluoxetine, escitalopram, and bupropion) prescribed in the management of depressive disorders have been associated with weight gain, insulin resistance, and broader disturbances in metabolic homeostasis.^7^ ^,^^8^ Accordingly, there is a strong rationale for systematically evaluating antidepressants with respect to metabolic safety, as accumulating evidence suggests that psychotropic exposure may contribute to alterations in metabolic parameters.^9^

Iatrogenic metabolic disturbances not only exacerbate the risk of developing cardiometabolic comorbidities in persons with depressive disorders but may also attenuate treatment response to monoaminergic antidepressants and adjunctive therapies.^10^ This bidirectional relationship further emphasizes the clinical importance of evaluating metabolic outcomes in the context of novel therapeutics.

Separately, findings from pivotal controlled studies with DXM/BUP and complementary open-label tolerability investigations suggest that DXM/BUP is not associated with clinically meaningful adverse effects on metabolic and anthropometric parameters.^2^ ^,^^11^ Nevertheless, further research is warranted as dextromethorphan’s activity at the σ-1 receptor, a target implicated in immunometabolic regulation, raises mechanistic questions about potential downstream effects on metabolic processes.^12^

Post-marketing pharmacovigilance offers an essential avenue for addressing the foregoing questions. The Food and Drug Administration Adverse Event Reporting System (FAERS) provides a large-scale repository of adverse event data to inform safety monitoring and clinical practice. To our knowledge, no prior FAERS-based study has specifically evaluated disproportionate reporting of metabolic adverse events associated with DXM/BUP. Herein, the present investigation aims to systematically evaluate FAERS reports of metabolic-related adverse events, including diabetes mellitus and hyperglycemia, in association with DXM/BUP.

## Method

### Comparator and adverse event selection and rationale

We synthesized post-marketing reports from the United States FAERS for DXM/BUP and comparator agents, including olanzapine (positive control), bupropion (clinically relevant comparator), and acetaminophen (reference comparator). Data were evaluated from the FDA approval date for each pharmacological agent (ie, DXM/BUP, 2022; olanzapine, 1996; bupropion, 1985; acetaminophen, 1969). These FDA-approved medications (ie, DXM/BUP, olanzapine, bupropion, and acetaminophen) were identified using their generic names, with trade names (eg, Auvelity, Zyprexa, Wellbutrin, and Tylenol) incorporated where necessary to ensure comprehensive capture of FAERS reports.

Comparator selection was guided by clinical relevance and pharmacovigilance context. Bupropion was included as a clinically relevant comparator within antidepressants, given its use in MDD and its role as a component of DXM/BUP. Olanzapine was included as a positive control due to its well-established association with metabolic adverse events and its use in mood disorders, including as adjunctive therapy in MDD. Lastly, acetaminophen was selected as a reference comparator, as it is a widely used agent with no well-established association with metabolic dysfunction, thereby providing a stable reference for background reporting patterns.

Each agent was assessed with respect to the reporting of diabetes mellitus and hyperglycemia from their FDA-approved date to April 26, 2026. For each drug-event pair, the number of reports with the event of interest (*n*) and without the event (non-cases) were extracted. Disproportionality was evaluated using reporting odds ratios (RORs), with corresponding 95% confidence intervals (CIs), Z-statistics, and *p*-values. Information component (IC) and IC_025_ values were calculated as exploratory measures (Tables 1 and 2). A sensitivity analysis was conducted using a restricted timeframe (January 1, 2022, to April 30, 2026) applied consistently across all agents to evaluate findings under a comparable reporting window (Table 2). Data extraction dates differed slightly due to sequential FAERS updates.

**Table 1.** DXM/BUP Metabolic Adverse Events (ie, Diabetes Mellitus and Hyperglycemia) Identified in the Food and Drug Administration’s Adverse Event Reporting System (FAERS), using Acetaminophen as the Reference Comparator, with Bupropion and Olanzapine Included for Contextual Comparison (All Available Years to April 2026) Table 1. long description.

Drug	Number of Cases (*n*)	Non-Cases (*N*)	ROR	Lower 95% CI	Upper 95% CI	Z Statistic	*P*-value	IC_025_ (comparator-based, exploratory)
Diabetes Mellitus								
Dextromethorphan/Bupropion	1	2,824	0.33	0.07	1.65	−1.35	0.18	−4.24
Bupropion	132	40,230	2.06	1.67	2.53	6.87	<0.001	0.38
Olanzapine	4,446	79,685	34.85	30.93	39.26	58.40	<0.001	1.48
Acetaminophen^a^	289	180,804	–	–	–	–	–	–
Hyperglycemia								
Dextromethorphan/Bupropion	0	2,825	0.10	0.01	1.67	−1.60	0.11	−7.45
Bupropion	43	40,319	0.63	0.46	0.87	−2.81	0.005	−1.11
Olanzapine	1,524	82,607	10.85	9.60	12.27	38.05	<0.001	1.25
Acetaminophen^a^	307	180,786	–	–	–	–	–	–

a Refers to the reference comparator.

Abbreviations: CI, Confidence interval; FAERS, Food and Drug Administration Adverse Event Reporting System; ROR, Reporting odds ratio; IC, Information component. IC and IC_025_ values were calculated using a comparator-based framework with a uniform continuity correction (+0.5) and are presented as supportive, exploratory metrics. Comparator-based IC_025_ values were directionally consistent with ROR estimates but attenuated in magnitude, reflecting methodological differences.

**Table 2.** DXM/BUP Metabolic Adverse Events (ie, Diabetes Mellitus and Hyperglycemia) Identified in the Food and Drug Administration’s Adverse Event Reporting System (FAERS), using Acetaminophen as the Reference Comparator, with Bupropion and Olanzapine Included for Contextual Comparison (January 1, 2022, to April 30, 2026) Table 2. long description.

Drug	Number of Cases (*n*)	Non-Cases (*N*)	ROR	Lower 95% CI	Upper 95% CI	*Z* Statistic	*P*-value	IC_025_ (comparator-based, exploratory)
Diabetes Mellitus								
Dextromethorphan/Bupropion	1	2,824	0.18	0.04	0.88	−2.11	0.035	−4.96
Bupropion	62	9,148	2.27	1.71	3.02	5.65	<0.001	0.36
Olanzapine	246	20,335	4.03	3.35	4.86	14.63	<0.001	0.90
Acetaminophen^a^	201	67,030	–	–	–	–	–	–
Hyperglycemia								
Dextromethorphan/Bupropion	0	2,825	0.11	0.01	1.80	−1.55	0.122	−7.33
Bupropion	2	9,208	0.17	0.05	0.60	−2.76	0.006	−4.34
Olanzapine	113	20,468	3.50	2.68	4.55	9.26	<0.001	0.66
Acetaminophen^a^	106	67,125	–	–	–	–	–	–

a Refers to the reference comparator.

Abbreviations: CI, Confidence interval; FAERS, Food and Drug Administration Adverse Event Reporting System; ROR, Reporting odds ratio; IC, Information component. IC and IC_025_ values were calculated using a comparator-based framework with a uniform continuity correction (+0.5) and are presented as supportive, exploratory metrics. Comparator-based IC_025_ values were directionally consistent with ROR estimates but attenuated in magnitude, reflecting methodological differences.

In addition, adverse events of interest were identified using MedDRA Preferred Terms corresponding to diabetes mellitus and hyperglycemia within the Metabolism and Nutrition Disorders system class, with case identification restricted to these terms to prioritize specificity.

### Statistical approach

Our analytic framework was informed by established pharmacovigilance methodologies that integrate frequentist (ROR) and IC-based disproportionality approaches, which have been widely applied in prior studies.^13^ ^–^^19^ This complementary strategy supports the identification of statistically robust signals while reducing the likelihood of spurious associations (Table 3).

**Table 3.** Calculations used for Investigating the Association Between Dextromethorphan/Bupropion and Metabolic Outcomes (ie, Diabetes Mellitus and Hyperglycemia) Table 3. long description.

	Target drug	Reference Comparator	Total
**AE of Interest**	**DE**	**dE**	**E**
**All Other AEs**	**De**	**de**	**e**
**Total**	**D**	**d**	**N**
*Here, ‘DE’ (a) refers to the count of reports in which the target drug is linked to the adverse event of interest. ‘De’ (b) corresponds to the number of reports of other adverse events involving the same target drug. ‘dE’ (c) denotes the number of reports of the adverse event of interest associated with reference comparator, while ‘de’ (d) indicates the number of reports of all other adverse events associated with reference comparator.*			
**Method**	**Equation**		
**ROR**	$\mathrm{ROR}=\left(a/c\right)/\left(b/d\right)=\mathrm{ad}/\mathrm{bc}$		
	$\mathrm{Se}\left(\text{InROR}\right)=\sqrt{(\frac{1}{a}+\frac{1}{b}+\frac{1}{c}+\frac{1}{d}})$		
	95% CI and Z-statistic calculations used for the reported ROR estimates: 95% CI = exp[ln(ROR) ± 1.96 × SE(ln ROR)] and Z = ln(ROR)/SE(ln ROR).			

First, RORs were calculated to evaluate disproportionality between reports with and without the adverse event of interest. In this study, ROR represents the relative reporting odds of metabolic adverse events associated with DXM/BUP and comparator agents relative to acetaminophen. Signal detection was interpreted in accordance with Evans’ criteria, which consider an ROR greater than 2.0, a chi-square statistic greater than 4.0, and at least three reported cases as indicative of a potential signal.^20^ These criteria were applied as a framework for interpretation rather than as a strict threshold for reporting.

In addition, a Haldane-Anscombe continuity correction was applied uniformly by adding 0.5 to all cells of each contingency table. This approach was implemented to enable estimation of RORs and corresponding CIs and to mitigate instability in sparse data settings. Next, corresponding 95% CIs were calculated to assess the precision of estimates. Statistical significance was defined as a 95% CI that excluded 1.0, consistent with *p* < 0.05.

As an exploratory measure, IC values and corresponding lower bounds (IC_025_) were calculated. Specifically, IC values were calculated to assess potential associations between the pharmacological agent of interest (ie, DXM/BUP) and adverse events. The IC compares the observed and expected reporting frequency for a given drug-event pair. An IC_025_ value greater than zero (IC_025_ > 0) was considered indicative of a potential signal, whereas an IC_025_ value less than or equal to zero (IC_025_ ≤ 0) indicates that no signal was observed. Given that IC metrics were derived within a comparator-based framework rather than the full FAERS database, these values are presented as supportive exploratory analyses.

All statistical analyses were conducted in R (version 4.5.1; R Foundation for Statistical Computing). Disproportionality metrics, including RORs and corresponding CIs, were calculated using standard formulas implemented directly in R. Data processing and visualization were performed using base R functions and commonly used packages, including dplyr and ggplot2.

## Results

Statistical analyses were conducted in R (version 4.5.1; R Foundation for Statistical Computing), with data collected from each pharmacological agent’s FDA approval date to April 26, 2026. For the primary analysis (all available years to April 26, 2026), a total of 2,825 adverse events were reported for DXM/BUP, including one report of diabetes mellitus and no reports of hyperglycemia. In comparison, 84,131 total adverse events were reported for olanzapine and 40,362 cases were reported for bupropion. Of the total olanzapine-related events, there were 4,446 diabetes mellitus and 1,524 hyperglycemia cases reported. For bupropion, there were 132 diabetes mellitus and 43 hyperglycemia adverse events reported. There was a total of 181,093 adverse event cases for acetaminophen, of which 289 cases were diabetes mellitus and 307 cases were hyperglycemia.

For the sensitivity analysis (January 1, 2022, to April 30, 2026), a total of 2,825 adverse event reports were identified for DXM/BUP, including one case of diabetes mellitus and no cases of hyperglycemia. In comparison, 20,581 adverse event reports were identified for olanzapine and 9,210 for bupropion. Among olanzapine reports, 246 cases of diabetes mellitus and 113 cases of hyperglycemia were identified. For bupropion, 62 cases of diabetes mellitus and 2 cases of hyperglycemia were reported. For acetaminophen, 67,231 adverse event cases were noted, including 201 cases of diabetes mellitus and 106 cases of hyperglycemia.

For each analysis, non-cases were defined as total adverse event reports excluding the event of interest. The RORs for DXM/BUP, olanzapine, and bupropion were calculated relative to acetaminophen as the reference comparator across both timeframes. While pairwise comparisons between agents may offer additional context, such comparisons were not conducted in the present analysis due to differences in clinical indication, baseline risk, and reporting behavior across drug classes, which may introduce bias in disproportionality estimates.

### Evaluation of diabetes mellitus reported events for dextromethorphan/bupropion

#### All available years to April 26, 2026

**Figure 1.**
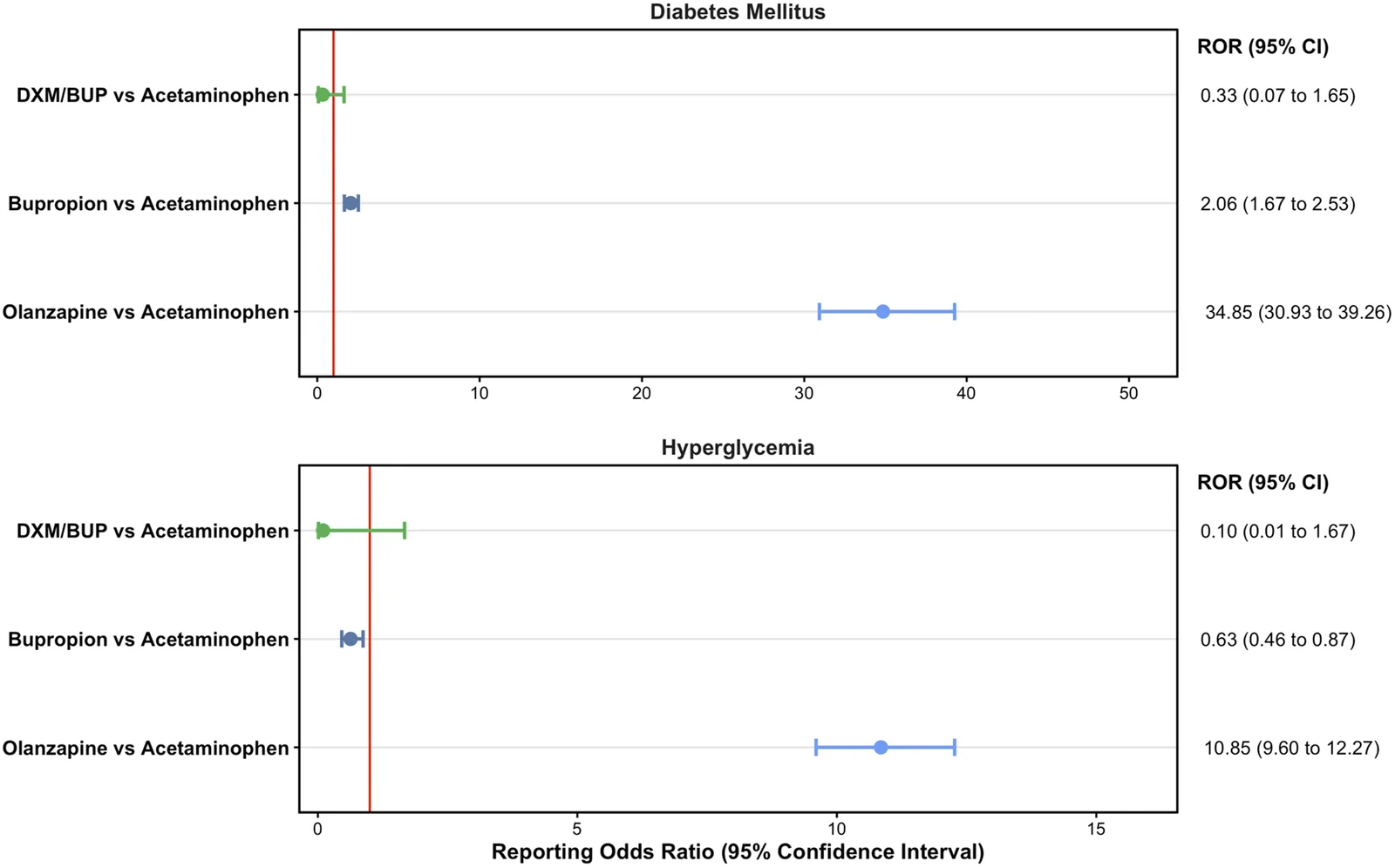
Forest plots of reporting odds ratio (ROR) for dextromethorphan/bupropion (DXM/BUP)-related metabolic disruptions (ie, diabetes mellitus and hyperglycemia) from agent’s start date to April 26, 2026. (top) Diabetes mellitus and (bottom) hyperglycemia reports for DXM/BUP, bupropion, and olanzapine compared with acetaminophen. Olanzapine was used as a positive control, whereas acetaminophen was used as a reference comparator. Reporting odds ratio (RORs) and 95% confidence intervals (CIs) are displayed, with the vertical red line indicating the null value (ROR = 1.0; acetaminophen). Estimates for DXM/BUP should be interpreted cautiously given limited case counts. Figure 1. long description.

With respect to diabetes mellitus across all years, DXM/BUP (ROR = 0.33, 95% CI [0.07, 1.65], *p* = 0.18) was not associated with disproportionate reporting relative to acetaminophen (Table 1; Figure 1). In contrast, olanzapine demonstrated a higher reporting (ROR = 34.85, 95% CI [30.93, 39.26], *p* < 0.001), while bupropion also exhibited elevated reporting (ROR = 2.06, 95% CI [1.67, 2.53], *p* < 0.001) relative to the reference comparator. Nonetheless, these findings should be interpreted in the context of differences in exposure and underlying populations across agents.

#### Restricted timeframe: January 1, 2022, to April 30, 2026

**Figure 2.**
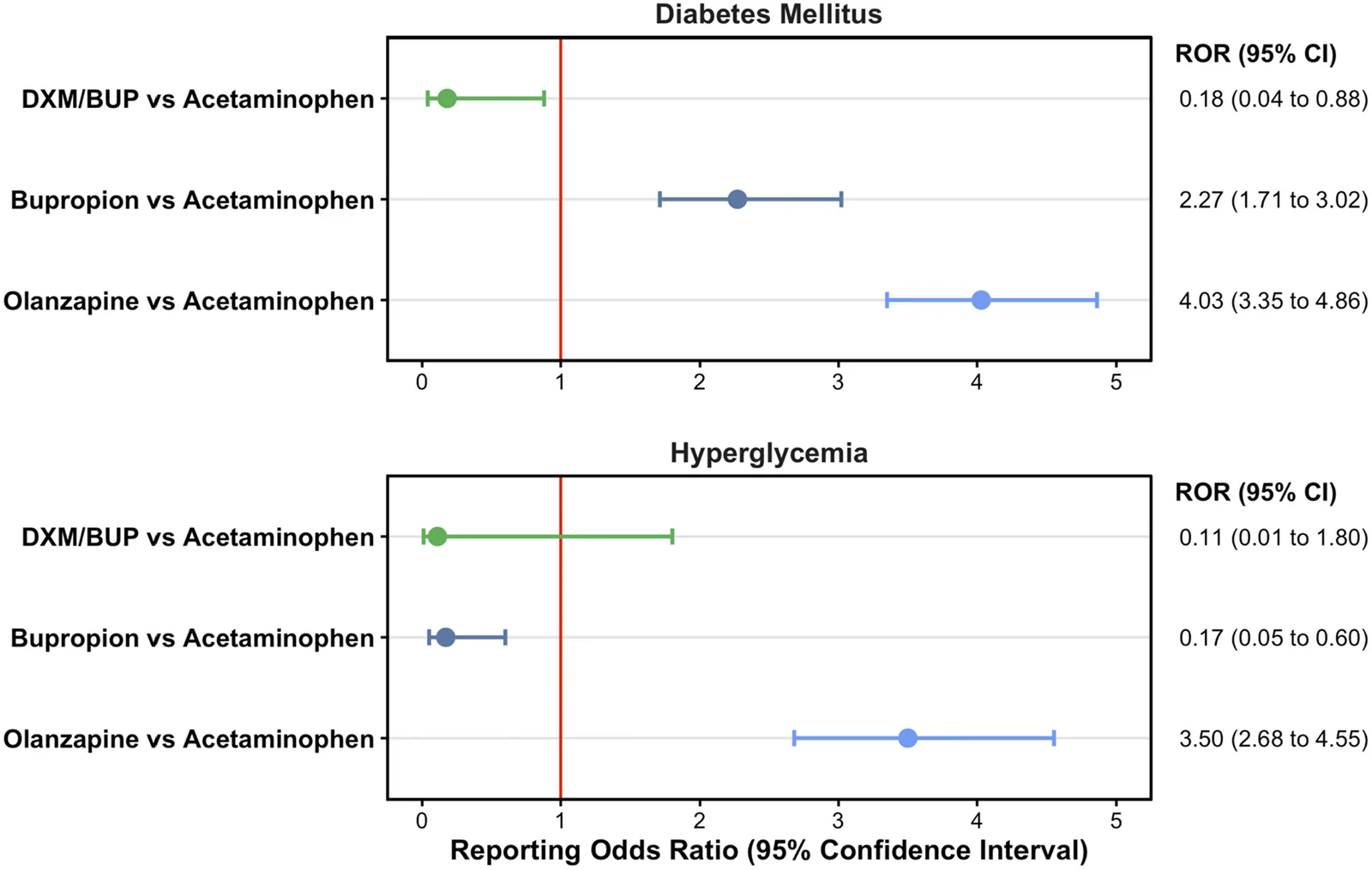
Forest plots of reporting odds ratio (ROR) for dextromethorphan/bupropion (DXM/BUP) related metabolic disruptions (ie, diabetes mellitus and hyperglycemia) from January 1, 2022 to April 30, 2026. (top) Diabetes mellitus and (bottom) hyperglycemia reports for DXM/BUP, bupropion, and olanzapine compared with acetaminophen. Olanzapine was used as a positive control, whereas acetaminophen was used as a reference comparator. Reporting odds ratio (RORs) and 95% confidence intervals (CIs) are shown, with the vertical red line indicating the null value (ROR = 1.0; acetaminophen). Estimates for DXM/BUP remain exploratory due to sparse reporting. Figure 2. long description.

Within the restricted timeframe, DXM/BUP (ROR = 0.18, 95% CI [0.04, 0.88], *p* = 0.035) demonstrated lower reporting of diabetes mellitus relative to acetaminophen, although this estimate should be interpreted cautiously given the limited number of cases (Table 2; Figure 2). Bupropion continued to show elevated reporting (ROR = 2.27, 95% CI [1.71, 3.02], *p* < 0.001), while olanzapine also demonstrated increased reporting (ROR = 4.03, 95% CI [3.35, 4.86], *p* < 0.001) relative to acetaminophen.

### Evaluation of hyperglycemia reported events for dextromethorphan/bupropion

#### All available years to April 26, 2026

DXM/BUP (ROR = 0.10, 95% CI [0.01, 1.67], *p* = 0.11) was not associated with disproportionate reporting of hyperglycemia compared to acetaminophen (Table 1; Figure 1). Olanzapine demonstrated increased reporting odds (ROR = 10.85, 95% CI [9.60, 12.27], *p* < 0.001), whereas bupropion was associated with lower reporting (ROR = 0.63, 95% CI [0.46, 0.87], *p* = 0.005) relative to the reference comparator. Nonetheless, the interpretation of these findings is limited by the small number of DXM/BUP cases and the resulting uncertainty in the estimates.

#### Restricted timeframe: January 1, 2022, to April 30, 2026

Within the restricted timeframe, DXM/BUP (ROR = 0.11, 95% CI [0.01, 1.80], *p* = 0.122) was also not associated with disproportionate reporting of hyperglycemia relative to acetaminophen (Table 2; Figure 2). Bupropion continued to demonstrate reduced reporting (ROR = 0.17, 95% CI [0.05, 0.60], *p* = 0.006), while olanzapine remained associated with increased reporting (ROR = 3.50, 95% CI [2.68, 4.55], *p* < 0.001). Findings were directionally consistent across the analyses.

## Discussion

Individuals living with MDD are at higher risk for metabolic disorders, including overweight or obesity.^5^ ^,^^21^ ^,^^22^ Several psychotropic agents commonly prescribed to individuals with MDD may exacerbate the intrinsic risk for metabolic disturbances.^23^ Separately, it could be speculated that, in addition to exacerbating risk for physical comorbidities (eg, cardiovascular disease), psychotropic agents that adversely affect metabolic parameters may contribute to difficulties in cognitive and reward functions.^24^ ^,^^25^ These considerations support the continued evaluation of metabolic safety when selecting treatments for MDD, particularly for individuals with elevated vulnerabilities.

Given that individuals with MDD are already at elevated baseline risk for obesity, cardiovascular disease, and metabolic disturbances, minimizing the prescription of agents with known metabolic liabilities is a crucial public health priority. Safer pharmacological options may reduce the compounding burden of physical comorbidities in this population, potentially lowering premature mortality, improving quality of life, and decreasing health care costs related to diabetes and cardiovascular disease. Furthermore, the possibility that glutamatergic modulation could exert neutral or favorable metabolic effects highlights the value of prioritizing such agents in treatment strategies and future research.

Results from pre-clinical, translational, and clinical pharmacological studies suggest that glutamatergic and σ-1 receptor mechanisms may intersect with metabolic pathways.^12^ ^,^^26^ For instance, interactions between NMDA/AMPA signaling and incretin or insulin pathways have been noted. These three effector systems (eg, incretin, insulin, and glutamate) have been implicated in metabolic homeostasis and neuroplastic and trophic processes critical to brain health.^27^ ^–^^29^ Notably, dextromethorphan has been reported to potentially augment incretin function in individuals with diabetes mellitus receiving incretin-based treatment.^29^ ^,^^30^ These mechanistic considerations should be regarded as hypotheses to guide future investigation rather than as interpretations supported by the present pharmacovigilance findings.

In the current analysis spanning each agent’s approval date to April 2026, DXM/BUP was not associated with disproportionate reporting signal for diabetes mellitus and hyperglycemia. Findings from the restricted 2022–2026 analysis were directionally consistent, supporting the primary analysis while accounting for differences in reporting duration across agents. However, this finding should not be interpreted as evidence that DXM/BUP is metabolically neutral or protective. On the contrary, the sparse case reporting for DXM/BUP suggests that FAERS exposure and reporting accumulation remain insufficient to support definitive conclusions. The observed trends are broadly consistent with separate lines of research, which suggests that DXM and BUP, as separate active pharmaceutical ingredients, are not associated with clinically meaningful adverse effects on metabolic and anthropometric parameters.^4^ ^,^^11^

Interestingly, bupropion alone demonstrated elevated reporting of diabetes mellitus across both analytical windows, whereas no corresponding signal was observed for DXM/BUP. Several explanations may account for this discordance. First, DXM/BUP remains a recently approved therapy with substantially lower cumulative exposure and adverse event reporting volume than bupropion, limiting statistical power to detect uncommon metabolic events. In addition, differences in indication, prescribing patterns, as well as varying patient characteristics may influence reporting behavior. For example, bupropion may be prescribed across diverse populations, including individuals receiving treatment for smoking cessation, who may differ in baseline cardiometabolic risk. Finally, disproportionality analyses evaluate reporting frequencies rather than incidence and remain vulnerable to confounding and reporting biases. Accordingly, the absence of a detectable DXM/BUP signal should not be interpreted as evidence of lower metabolic risk relative to bupropion.

Several methodological and data limitations affect the inferences and interpretations of our results. For instance, the small number of reported events associated with DXM/BUP may reflect insufficient case accumulation within the FAERS database, contributing to wide CIs and limiting statistical precision. Consistent with this, DXM/BUP did not meet the minimum case threshold specified by Evans’ criteria, which further constrains interpretability and precludes classification as a pharmacovigilance signal.

In addition, spontaneous reporting systems such as FAERS are subject to well-recognized limitations, including incomplete case information, potential duplicate reporting, lack of case verification, and reporting bias. Differences in time since approval across agents may also affect reporting patterns, particularly for outcomes with longer latency such as diabetes mellitus. Notably, the restricted 2022–2026 analysis was intended to provide a common calendar-period comparison and account for contemporaneous reporting practices. However, this approach does not fully control for differential time-since-approval effects, including the Weber effect and the potentially prolonged latency between drug exposure and diabetes diagnosis. While the inclusion of clinically relevant and contextual comparators was intended to support interpretation of reporting patterns, differences in clinical indication, underlying population characteristics, and cumulative exposure across agents remain as considerations.

Lastly, the use of selected MedDRA Preferred Terms may underestimate the total burden of metabolic adverse events, as related conditions captured under broader or alternative coding terms (eg, higher-level groupings) were not included. For example, related events such as insulin resistance, metabolic syndrome, weight gain, or impaired glucose tolerance can be incorporated to expand MedDRA queries and provide a more comprehensive assessment. Future analyses incorporating expanded MedDRA queries may provide a more thorough evaluation.

Given these considerations, and from a pharmacovigilance perspective, the current findings from the primary and exploratory analyses should be interpreted cautiously. Distinctions in clinical indication, patient populations, duration of exposure, and reporting behavior across comparator agents limit direct metabolic risk comparisons. Cumulatively, these results should not be used to inform comparative antidepressant selection or as evidence of relative safety. Instead, they emphasize the need for continued monitoring and evaluation of metabolic outcomes as real-world exposure to DXM/BUP increases.

## Conclusion

Ultimately, we did not identify disproportionate reporting of select metabolic adverse events associated with DXM/BUP in the primary and sensitivity FAERS analyses. While these findings may be considered cautiously reassuring, they should be interpreted with caution, given the inherent limitations of pharmacovigilance data, including potential underreporting, incomplete case information, and lack of causal inference. Importantly, individuals with MDD remain at heightened risk for metabolic comorbidities, emphasizing the need for treatment strategies that address both psychiatric and physical health outcomes in an integrated manner. Within this context, the present findings do not permit definitive conclusions regarding the metabolic safety of DXM/BUP. Continued pharmacovigilance, analysis using clinically relevant and diverse antidepressant comparators, and prospective studies with adequate sample size and metabolic outcome measurement are warranted to better characterize the metabolic safety profile of DXM/BUP.

## Data Availability

The data analyzed in this study were obtained from the U.S. Food and Drug Administration Adverse Event Reporting System (FAERS), which is publicly available. The processed dataset is available from the corresponding author upon reasonable request.

## Long descriptions

### Table 1. Long description

The table contains 9 columns: Drug, Number of Cases (n), Non-Cases (N), R O R, Lower 95% C I, Upper 95% C I, Z Statistic, P-value, and I C sub 025 (comparator-based, exploratory).

Section 1: Diabetes Mellitus

* Dextromethorphan/Bupropion: 1 case, 2,824 non-cases, R O R 0.33, C I 0.07 to 1.65, Z -1.35, P 0.18, I C sub 025 -4.24.

* Bupropion: 132 cases, 40,230 non-cases, R O R 2.06, C I 1.67 to 2.53, Z 6.87, P less than 0.001, I C sub 025 0.38.

* Olanzapine: 4,446 cases, 79,685 non-cases, R O R 34.85, C I 30.93 to 39.26, Z 58.40, P less than 0.001, I C sub 025 1.48.

* Acetaminophen (Reference): 289 cases, 180,804 non-cases.

Section 2: Hyperglycemia

* Dextromethorphan/Bupropion: 0 cases, 2,825 non-cases, R O R 0.10, C I 0.01 to 1.67, Z -1.60, P 0.11, I C sub 025 -7.45.

* Bupropion: 43 cases, 40,319 non-cases, R O R 0.63, C I 0.46 to 0.87, Z -2.81, P 0.005, I C sub 025 -1.11.

* Olanzapine: 1,524 cases, 82,607 non-cases, R O R 10.85, C I 9.60 to 12.27, Z 38.05, P less than 0.001, I C sub 025 1.25.

* Acetaminophen (Reference): 307 cases, 180,786 non-cases.

Navigate back to Table 1..

### Table 2. Long description

The table contains 9 columns: Drug, Number of Cases (n), Non-Cases (N), R O R, Lower 95% C I, Upper 95% C I, Z Statistic, P-value, and I C sub 025 (comparator-based, exploratory).

Section 1: Diabetes Mellitus

* Dextromethorphan/Bupropion: 1 case, 2,824 non-cases, R O R 0.18, C I 0.04 to 0.88, Z -2.11, P 0.035, I C sub 025 -4.96.

* Bupropion: 62 cases, 9,148 non-cases, R O R 2.27, C I 1.71 to 3.02, Z 5.65, P less than 0.001, I C sub 025 0.36.

* Olanzapine: 246 cases, 20,335 non-cases, R O R 4.03, C I 3.35 to 4.86, Z 14.63, P less than 0.001, I C sub 025 0.90.

* Acetaminophen (Reference): 201 cases, 67,030 non-cases.

Section 2: Hyperglycemia

* Dextromethorphan/Bupropion: 0 cases, 2,825 non-cases, R O R 0.11, C I 0.01 to 1.80, Z -1.55, P 0.122, I C sub 025 -7.33.

* Bupropion: 2 cases, 9,208 non-cases, R O R 0.17, C I 0.05 to 0.60, Z -2.76, P 0.006, I C sub 025 -4.34.

* Olanzapine: 113 cases, 20,468 non-cases, R O R 3.50, C I 2.68 to 4.55, Z 9.26, P less than 0.001, I C sub 025 0.66.

* Acetaminophen (Reference): 106 cases, 67,125 non-cases.

Navigate back to Table 2..

### Table 3. Long description

The table is divided into two main sections.

Section 1: Contingency Matrix

* Rows: A E of Interest, All Other A Es, and Total.

* Columns: Target drug (D E, D e, D), All other drugs (d E, d e, d), and Total (E, e, N).

* Definitions: D E is the count of reports linking the target drug to the adverse event of interest. D e is other adverse events for the target drug. d E is the adverse event of interest for all other drugs. d e is all other adverse events for all other drugs.

Section 2: Methods and Equations

* R O R (Reporting Odds Ratio): R O R equals (a / c) / (b / d) which simplifies to a d / b c. The standard error S e (l n R O R) equals the square root of (1 / a plus 1 / b plus 1 / c plus 1 / d).

Navigate back to Table 3..

### Figure 1. Long description

Two stacked forest plots. Both plots feature a horizontal x-axis for Reporting Odds Ratio (R O R) with 95 percent Confidence Intervals (C I) and a vertical red line at R O R equals 1.0 representing the null value (acetaminophen).

Top Panel: Diabetes Mellitus. The x-axis ranges from 0 to 50.

* D X M forward slash B U P vs Acetaminophen: Green dot at 0.33 (C I 0.07 to 1.65), crossing the null line.

* Bupropion vs Acetaminophen: Dark blue dot at 2.06 (C I 1.67 to 2.53), positioned to the right of the null line.

* Olanzapine vs Acetaminophen: Light blue dot at 34.85 (C I 30.93 to 39.26), far to the right of the null line.

Bottom Panel: Hyperglycemia. The x-axis ranges from 0 to 15.

* D X M forward slash B U P vs Acetaminophen: Green dot at 0.10 (C I 0.01 to 1.67), crossing the null line.

* Bupropion vs Acetaminophen: Dark blue dot at 0.63 (C I 0.46 to 0.87), positioned to the left of the null line.

* Olanzapine vs Acetaminophen: Light blue dot at 10.85 (C I 9.60 to 12.27), far to the right of the null line.

Navigate back to Figure 1..

### Figure 2. Long description

Two stacked forest plots. The x-axis for both represents the Reporting Odds Ratio, abbreviated R O R, with 95 percent Confidence Intervals, abbreviated C I, ranging from 0 to 5. A vertical red line at R O R equals 1.0 indicates the null value for the reference agent, acetaminophen.

Top Panel: Diabetes Mellitus.

* D X M forward slash B U P versus Acetaminophen: A green dot at 0.18 with a narrow horizontal bar from 0.04 to 0.88, positioned to the left of the null line.

* Bupropion versus Acetaminophen: A dark blue dot at 2.27 with a bar from 1.71 to 3.02, positioned to the right of the null line.

* Olanzapine versus Acetaminophen: A light blue dot at 4.03 with a bar from 3.35 to 4.86, positioned furthest to the right.

Bottom Panel: Hyperglycemia.

* D X M forward slash B U P versus Acetaminophen: A green dot at 0.11 with a wide horizontal bar from 0.01 to 1.80, crossing the null line.

* Bupropion versus Acetaminophen: A dark blue dot at 0.17 with a narrow bar from 0.05 to 0.60, positioned to the left of the null line.

* Olanzapine versus Acetaminophen: A light blue dot at 3.50 with a bar from 2.68 to 4.55, positioned to the right of the null line.

Navigate back to Figure 2..
